# Early Experience With Robotic Approaches to Inflammatory Bowel Disease Surgery: A Single-Center Study

**DOI:** 10.7759/cureus.99403

**Published:** 2025-12-16

**Authors:** Vinayak P Thattaruparambil, Nur Jenny, Rebecca Kay, Ann B Konkoth, Neena Randhawa

**Affiliations:** 1 Colorectal Surgery, Newcastle upon Tyne Hospitals NHS Foundation Trust, Newcastle upon Tyne, GBR; 2 Biostatistics, Population Health Sciences Institute, Newcastle University, Newcastle upon Tyne, GBR; 3 Colorectal Surgery, Newcastle upon Tyne Hospitals NHS Foundation Trust, Newcastle Upon Tyne, GBR

**Keywords:** inflammatory bowel diseases (ibd), laparoscopic surgery, minimally invasive surgery, robotic surgery, surgical outcomes

## Abstract

Background

Understanding the surgical outcomes following surgery for inflammatory bowel disease (IBD) is crucial for proper consent and informed decision-making. Contemporary recommendations to guide IBD surgical intervention now favor a minimally invasive approach, if possible. We present surgical outcomes following the introduction of robotic minimally invasive surgery (MIS) for IBD in a tertiary center.

Aim

Robotic approaches to colorectal resections are now becoming routine for colorectal cancer surgery but use in IBD is still not currently mainstream. Here, we describe our initial experience with robotic IBD surgery and compare 30-day surgical outcomes to laparoscopic and open approaches.

Methods

A single tertiary referral center experience of all patients over 16 years of age undergoing surgery for IBD between 2020 and 2023 was analyzed. This retrospective review of all prospectively gathered surgical data utilized the hospital database and prospectively recorded ERAS (Enhanced Recovery After Surgery) records detailing patient recovery and complications. For the statistical calculations, we used ANOVA for overall data and Tukey's HSD for comparing each cohort.

Results

A total of 140 cases were analyzed, showing mean procedure time in minutes across robotic, laparoscopic, and open approaches (p=0.997): colectomies (268, 282, 265), panproctocolectomies (380, 425, 382), proctectomies (315, 259, 228), and small bowel resections (161, 165, 202). There was zero mortality across all groups. For robotic, laparoscopic, and open approaches, the major complications (Clavien-Dindo classification 3+) rates were 5.4%, 8%, and 3.5% (p=0.686), respectively, and the readmission rates were 8.10%, 9.33%, and 21.42%, respectively. The mean post-operative stays in days (p=0.164) were as follows: colectomies (6.70, 8.45, 10.33), panproctocolectomy (8.40, 8.92, 10.29), completion proctectomy (6.00, 5.00, 8.20), and ileocecal/small bowel resection (6.42, 6.46, 6.80). The costs for patients undergoing robotic surgery were higher (p=0.11).

Conclusion

This early data demonstrate real-world experience of the introduction of robotic surgical techniques for colorectal resection. Robotic surgical outcomes demonstrate equivalence with the laparoscopic outcomes and are improved compared to open procedures in most metrics but are more expensive.

## Introduction

Surgical treatment is an important component in inflammatory bowel disease (IBD) since up to 80% of Crohn’s disease (CD) and 30% of ulcerative colitis (UC) patients may require bowel resection [1]. Minimally invasive surgery (MIS) has been utilized in the treatment of IBD for more than 20 years [1,2]. An important advantage of MIS is the decreased effect of surgery on the inflammatory process [1]. IBD patients mostly have surgery during acute inflammation, which can exaggerate the effects of trauma caused by surgery [1]. Lower adhesion formation after MIS is also a benefit given that upto 35% of CD patients undergoing surgery will need a re-operation in the next 10 years and a multiple-step surgery for total colectomy in UC patients [1,3]. This lower surgical trauma of MIS has a favorable effect on the immune system, which is a key part in the pathogenesis of IBD and therefore causes a faster recovery [4,5]. A factor that significantly impacts reoperation in CD patients is the presence of a stoma. As shown by some studies, ileostomy creation was significantly higher in the OA cohort despite comparable comorbidities and nutritional status [6]. In the fragile cohort of IBD patients, the introduction of MIS has brought about improved outcomes and quality of care [1].

Robotic surgery has evolved as an effective and precise addition to MIS utilizing micro instruments, high-quality cameras, and augmented reality based on laparoscopic principles, improving patient safety and reducing post-operative complications and early return to routine life [1,2]. Robotic approach (RA) in colorectal surgery is becoming increasingly popular globally due to its improved dexterity, tremor-free movements, and other benefits over laparoscopy [1,2]. Robotic surgery combines the benefits of MIS with better technical agility. Lower rectal resections, especially relevant in UC, can be a technical challenge by conventional laparoscopic approach (LA) due to limited space, increasing the chances of complications. RA offers high-definition three-dimensional visualization, stable camera platform, wristed instruments, immunofluorescence capability, and tremor reduction [7], which can reduce the risks of pelvic nerve damage, resulting in better quality of life outcomes, shorter bowel function time, and decreased readmission rates. Many studies looking at RA in IBD have shown some advantages over LA. Robotic ileocolic resection for CD has shorter post-operative ileus, decreased conversion to open and overall reduced complication rates [8]. RA in proctocolectomy and ileal pouch anal anastomosis in UC patients has demonstrated reduced estimated blood loss, complications, and readmission rates as compared to LA [9]. In this study, we outline our early experience with the introduction of robotic experience in a tertiary IBD center and analyze cost and complication rates and impact on discharge and readmissions.

## Materials and methods

Data collection

This is a retrospective single-center analysis of all patients who underwent robotic surgery with an underlying pathologically confirmed diagnosis of IBD. All patients operated on at the Newcastle Upon Tyne Trust Hospitals between January 1, 2020, to April 1, 2023, were included if they had a diagnosis of IBD and were over the age of 16 years. Only patients who underwent panproctocolectomies, colectomies, proctectomies, and small bowel resections for IBD surgery were included. Patients underwent robotic, laparoscopic, or open approach based on if the procedure was done in an elective or acute setting. The approach was also decided by the patient's BMI and surgeon's preference or expertise. A retrospective review of surgical data was undertaken to identify relevant patients from the hospital database (Powerchart™ and Surginet™) and our in-house Enhanced Recovery After Surgery (ERAS) records. An excel database included demographic recordings of age, gender (male/female), disease (UC/CD), type of surgery (panproctocolectomies, colectomies, proctectomies, and small bowel resections) and modality (RA/LA/open approach [OA]), duration of surgery, conversion rates from intended methodology, post-operative stay, 30-day Clavien-Dindo complication rates (3+), 30-day readmission rates, mortality, and costs of care. The cost of the care was calculated by adding theater costs including consumables for each procedure and cost of stay in the ward for each day. The trust Caldicott Guardian approval was gained, and the study was registered (Project Number: 14015) as part of audit process within the hospital comparing different surgical approaches.

Statistical analysis

For the statistical calculations, we used ANOVA for overall data and Tukey's HSD (honestly significant difference) for comparing each cohort as a post-hoc test to look for significance. We also used the Kruskal-Wallis rank sum test to check the association between treatment groups and Clavien-Dindo 3+ complications as this was non-parametric and did not follow normal distribution. Both ANOVA/Tukey's and Kruskal-Wallis tests had to be used as continuous variables were checked for normality and as cohort sizes in some procedure-type subgroups were small, which limited the power of subgroup comparisons.

## Results

We had a total of 140 patients who fit our inclusion criteria, of whom 72 (52%) were males. The median age was 39 years (IQR=29-56 years; range: 17-81 years). There were 94 (67%) patients with CD and 46 patients with UC. In terms of procedure, 45 patients underwent colectomy, and 25 patients had a panproctocolectomy and proctectomy each. Also, 45 patients underwent small bowel resections for CD, and RA was used in 37 (26%) of these patients. Laparoscopic procedure, which is the current gold standard, was performed in 75 of them. OA was required in 28 patients (Table 1).

**Table 1:** Demographics of patients with the pathology and procedures underwent. The table encompasses our sample size with their demographic features including median age, gender, and diagnosed pathology for each of the procedures. It also shows how many patients underwent a robotic, laparoscopic, or open approach for each procedure. F=female; M; male; CD=Crohn’s disease; UC=ulcerative colitis; RA=robotic approach; LA=laparoscopic approach; OA=open approach

Age in years (median)	F	M	CD	UC	Procedure	RA	LA	OA	Total
36	24	21	26	19	Colectomies	10	29	6	45
51	13	12	15	10	Panproctocolectomies	5	13	7	25
39	11	14	8	17	Proctectomy	10	10	5	25
41.5	20	25	45	0	Small bowel Resections	12	23	10	45
39	68	72	94	46	Total	37	75	28	140

Our mean procedure times for robotic colectomies, panproctocolectomies, proctectomies, and small bowel resections were 268.20, 380.40, 315.50, and 161.67 minutes, respectively. In our study, we found that the procedure time of robotic cases was better for colectomies, panproctocolectomies, and small bowel resections and was comparable for proctectomies against laparoscopic surgery. Open colectomies and proctectomies finished faster than other modalities. It was comparable to RA in panproctocolectomy but higher for small bowel resections. The procedure time of the robotic procedures was also influenced by the learning curve, surgeon bias, and expertise (Table 2).

**Table 2:** Average procedure time breakdown between the different procedures and modalities. RA=robotic approach; LA=laparoscopic approach; OA=open approach

Procedure	Average procedure time (minutes) (p=0.997)		
	RA	LA	OA
Colectomy	268.20	282.24	265.17
Panproctocolectomy	380.40	425.54	382.71
Proctectomy	315.50	259.50	228.60
Small bowel resection	161.67	170.17	202.30

We had a very similar conversion rate to open in both robotic 10.8% (n=4/37) and laparoscopic 10.5% (n=8/76) repairs.

The ileus rates were highest in the RA (24.3%), followed by OA (17.8%) and LA (12%). The readmission rates were lowest in robotic procedures (8.10%), followed by laparoscopic (9.33%) and open (17.85%) procedures (Table 3).

**Table 3:** Post-operative ileus and readmission rates between the different approaches of surgery. The table demonstrates post-operative ileus and readmission rates between the different approaches of surgery. The rates were calculated by taking the number of patients who had post-operative ileus or a readmission as the numerator. The total number of patients who had the same approach, i.e., robotic, laparoscopic, or open, was taken as the denominator. Therefore n=rate of ileus/readmission.

Procedures	Ileus		Readmission in 30 days (p=0.174)	
	Rates (n)	Percentage (%)	Rates (n)	Percentage (%)
Robotic	9/37	24.32	3/37	8.10
Laparoscopic	9/75	12	7/75	9.33
Open	5/28	17.85	5/28	17.85

**Figure 1:**
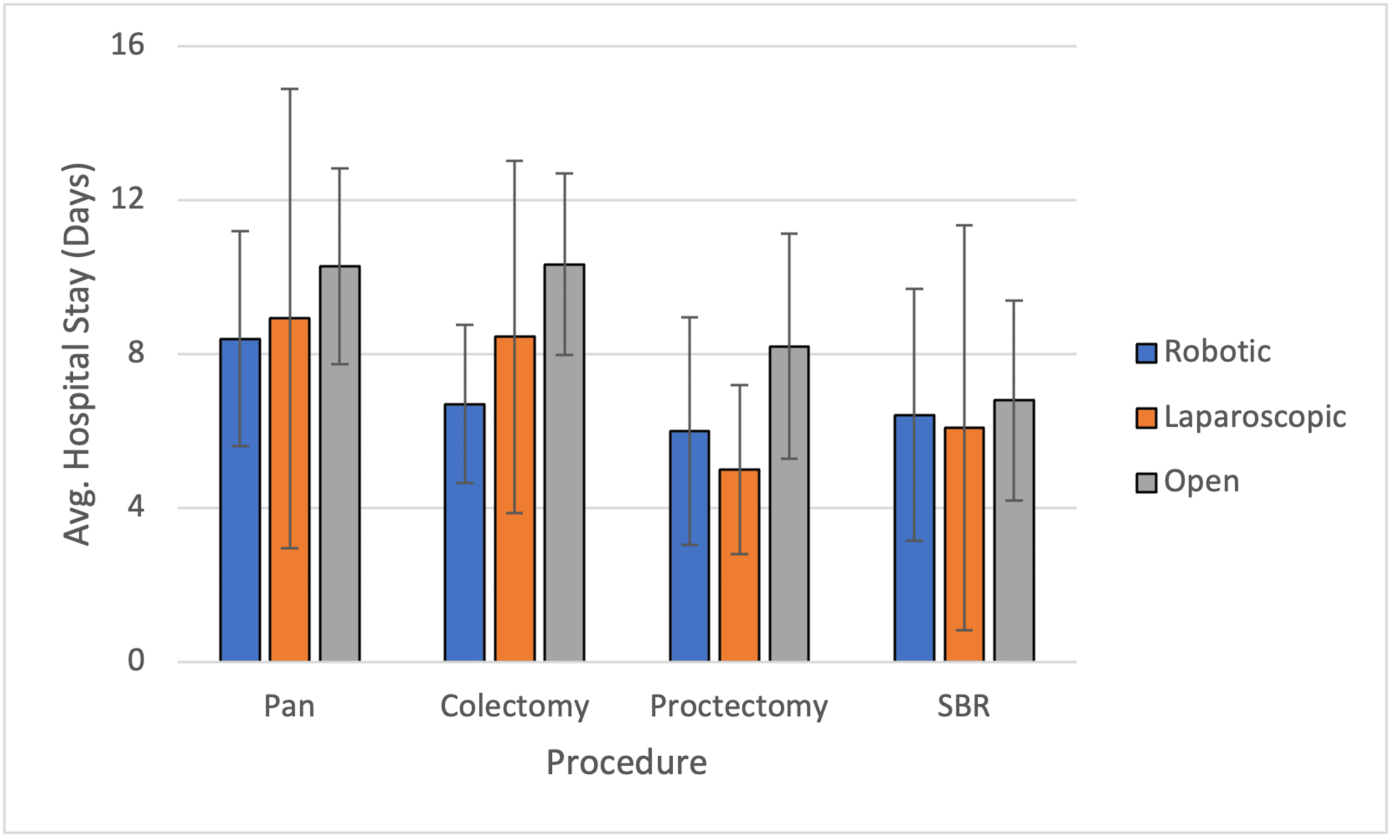
Average hospital stay plotted against the procedures for different modalities. Bar graph plots average hospital stay in days with standard deviations plotted against the procedures for different modalities. Pan=panproctocolectomy; SBR=small bowel resection

**Figure 2:**
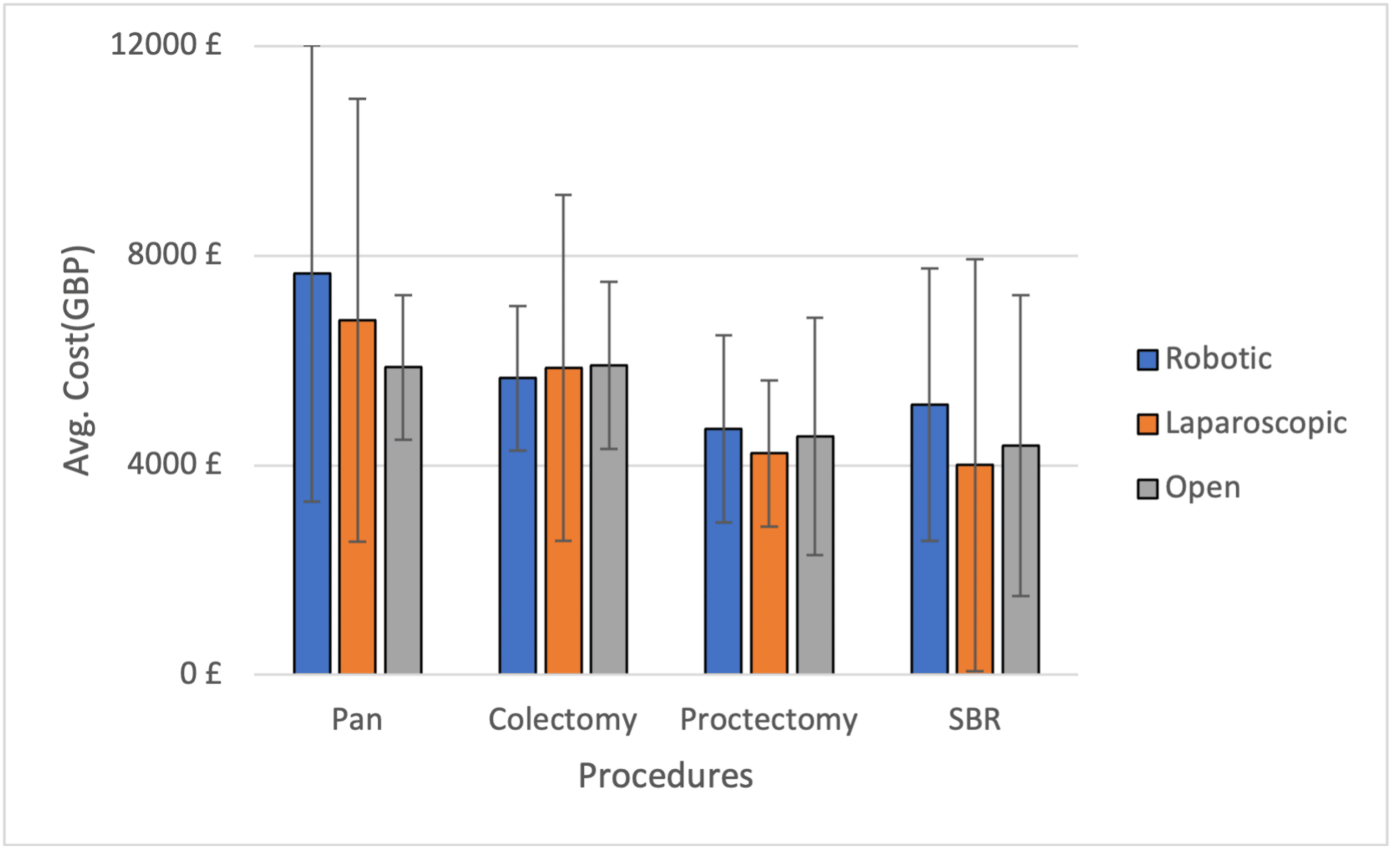
Average cost plotted against the procedures for different modalities. Bar graph denotes the average cost in GBP with standard deviations plotted against the procedures for different modalities. GBP=Great Britain pound; Pan=panproctocolectomy; SBR=small bowel resection

The average hospital stay after a robotic procedure was lower for all procedures except proctectomies, where it was almost comparable with laparoscopy. The average stay was longer in all open procedures (Figure 1). All robotic procedures were more expensive than their counterpart modalities except colectomies, which were cheaper than laparoscopy and open procedures. The total cost for each procedure was calculated by combining the costs for the theater consumables with the cost of stay in the ward per day (Figure 2, Table 4).

**Table 4:** Average hospital stays and cost between the different surgical procedures and their approaches. The table shows average hospital stays in days and cost in GBP between the different surgical procedures and their approaches. RA=robotic approach; LA=laparoscopic approach; OA=open approach; GBP=Great Britain pound

Procedures	Average hospital stay (days) (p=0.164)			Average total cost (GBP) (p=0.11)		
	RA	LA	OA	RA	LA	OA
Colectomies	6.70	8.45	10.33	£5661.65	£5855.23	£5907.59
Panproctocolectomies	8.40	8.92	10.29	£7655.57	£6764.59	£5868.53
Proctectomies	6.00	5.00	8.20	£4693.32	£4229.09	£4549.91
Small bowel resections	6.42	6.46	6.80	£5160.97	£4003.30	£4382.03

The post-operative complications were classified into Clavien-Dindo categories 1 to 5. There was no mortality across any groups, and none of them had a category 5 complication. Only one patient in the LA group had a category 4 complication. Surprisingly, open procedures had the least number of CD 3+ complications at 3.5%. Robotic procedures had lesser (5.4%) CD 3+ complication rates than the laparoscopic ones (8%) (Table 5).

**Table 5:** Post-operative complications classified as per Clavien-Dindo for different approaches. The table demonstrates post-operative complications classified as per Clavien-Dindo for different approaches. Clavien-Dindo classification 1-5 for robotic, laparoscopic, and open approach are shown above. Only grades 3 and above were used for calculating complication rates. n=number of patients having Clavien- Dindo 3 and above complication as the numerator. The denominator is the total number of patients in each group of approach.

Procedures	Clavien-Dindo 3+ complication (p=0.686)						
	1	2	3	4	5	Grade 3 + (n)	Percentage (%)
Robotic	7	4	2	0	0	2/37	5.4
Laparoscopic	8	13	5	1	0	6/75	8
Open	5	5	1	0	0	1/28	3.5

The statistical analysis utilized ANOVA tests for procedure time, hospital stay, and total costs between the treatment groups and did not show any statistical significance. The Kruskal-Wallis rank sum test was used to check the association between the treatment groups and modalities, and treatment groups and Clavien-Dindo 3+ complication rates did indicate any statistical significance either. The chi-squared test was used to check the association between readmission and the surgical approaches, which showed no statistical significance (Table 6).

**Table 6:** Statistical tests with their respective p-values. ANOVA=analysis of variance

Tests	P-value (significant if p≤0.05)
ANOVA test between treatment groups and procedure time	0.997
ANOVA test between treatment groups and hospital stay	0.164
ANOVA test between treatment groups and total cost	0.11
Kruskal-Wallis rank sum test to check the association between treatment groups and types of surgery	0.8411
Kruskal-Wallis rank sum test to check the association between treatment groups and Clavien-Dindo 3+ complications	0.2167
Chi-square test to check association between readmission and groups (robotic and open)	0.239
Chi-square test to check association between readmission and groups (robotic and laparoscopic)	1.000
Chi-square test to check association between readmission and groups (laparoscopic and open)	0.1898
Chi-square test to check association between readmission and all 3 groups	0.1743

Tukey's HSD test was used as a post-hoc statistical test after ANOVA to identify statistical significance of the mean values of procedure time, hospital stay, and total cost between different surgical modalities, which did not yield any significance (Table 7).

**Table 7:** Tukey's HSD test and p-values between the cohorts.

	P-values (significant if p≤0.05)
Tukey's test between procedure time and treatments	
Open and laparoscopy	0.99
Robotic and laparoscopy	0.99
Robotic and open	0.99
Tukey's test between hospital stays and treatments	
Open and laparoscopy	0.34
Robotic and laparoscopy	0.69
Robotic and open	0.14
Tukey's test between total cost and treatments	
Open and laparoscopy	0.9
Robotic and laparoscopy	0.095
Robotic and open	0.34

Linear regression was used to quantify the relationship between the modalities and the procedure time, hospital stay, and theater costs. It showed that the theater cost for open treatment was on an average £340.77 lesser than the laparoscopic procedure and that the robotic procedure was £784.13 higher than the laparoscopic procedure, yielding significance. The procedure time and hospital stay did not yield any significance (Table 8).

**Table 8:** Linear regression analysis.

Linear regression to see the relation between	P-values	Interpretation
Procedure time and treatment	0.9969	Insignificant
Hospital stays and treatment	0.1641	Insignificant
Theater cost and treatment	<2.2e-16	The theater cost for open treatment was on an average £340.77 lesser than the laparoscopic procedure. Robotic procedure was £784.13 higher than the laparoscopic procedure, and the results were quite significant.

## Discussion

Robotic surgery has overcome many of the technical issues of laparoscopy. Many single and multicenter studies have shown that robotic ileocolic, colon, and rectal resections are feasible and safe [8]. Even though statistically insignificant and more expensive, our results showed that RA in IBD surgery is safe and comparable with gold standard LA. A staged panproctocolectomy is the procedure of choice to eliminate disease burden and offer cure in UC [10]. Even though the commonest surgery in CD patients is an ileocecal resection, depending on the disease location and mapping, they may need operations in the small bowel, colon, or perineum [10].

In this study, mean procedure times for robotic colectomies, panproctocolectomies, proctectomies, and small bowel resections were 268.20, 380.40, 315.50, and 161.67 minutes, respectively. Multiple studies comparing robotic to laparoscopic surgery in IBD revealed a significantly longer procedure time for the robotic arm [10,11]. On the contrary, the surgical software database recordings showed better procedure times for robotic cases in colectomies, panproctocolectomies, and small bowel resections and were comparable for proctectomies with laparoscopic surgery in our study. Laparoscopic proctectomies finished faster than robotic by 30-45 minutes on average and had cost implications, but this could also be due to disease severity, the learning curve of the new modality, and the surgeon's expertise [10].

In this study, the conversion rate for robotic to open was 10.81%. It was comparable with the conversion rate for laparoscopic to open, which was 10.6%. A study looking at ileocolic resections in CD showed lower conversion rates in the RA [8]. Systematic reviews looking at conversion rates showed statistically insignificant but reduced conversion rates in RA [10,12].

The study showed that the mean length of stay was 6.70 (SD=2.05), 8.40 (SD=2.8), 6.00 (SD=2.96), and 6.42 (SD=3.27) days for robotic colectomies, panproctocolectomies, proctectomies, and small bowel resections, respectively. This was better than or comparable to the laparoscopic and open cases in the study. This is similar to published studies, which showed favorable hospital stay after a robotic procedure, although this was not statistically significant as compared to other modalities [10,12].

To address bowel functional outcomes, we only looked at ileus rates, which were comparable in RA and OA but better in LA. In a recent systematic review looking at seven studies comparing minimal invasive operations in IBD, the post-operative ileus rates were comparable [10].

The study identified complication rates for IBD colorectal robotic, laparoscopic, and open surgeries, which were 35.13%, 36.84%, and 39.28%, respectively. Considering Clavien-Dindo classification grade 3 and above, the complication rates were 5.4%, 8%, and 3.5% respectively, which is an appreciable difference in major complications when comparing the robotic group with the laparoscopic group but insignificant like other studies [12]. A meta-analysis conducted by Renshaw et al. across eight studies looking at robotic IBD surgery found 54% of complications across UK, whereas we had a total percentage of 35.13%. The same review claimed a 20% for Clavien-Dindo III-IV complications, whereas we had 5.4% for the same [11]. The 30-day mortality rate was 0% for all modalities of surgery, which is consistent with studies looking at minimal invasive surgery in IBD [10,11].

A systemic review of eight studies in robotic surgery in IBD across the UK reported a readmission rate of 24.7% [11]. One of those studies comparing RA and LA showed better rates in the earlier group but no statistical significance [12]. Overall, 8.10% of our robotic patients were readmitted in the first 30 days as opposed to 9.21% and 21.42% in the laparoscopic and open groups, respectively. Another systematic review looking at four studies reported a readmission rate of 12.9% in the robotic group as compared to 20.7 in the laparoscopic group, which was not statistically significant [10]. Our study showed a lower rate but not statistically significant.

No studies compared the cost-effectiveness between robotic and traditional approaches for IBD colorectal robotic surgeries. On calculating the average total cost, which included approximate theater costs and stay in ward, we found that the robotic procedure costs were almost double in some procedures, and our linear regression showed it was significantly higher as compared to LA. Given that robotic surgery is a relatively new modality at our center, it has procured added costs on training, cost of new equipment, and the cost of robotic biomedical equipment. The learning curve is also a factor in determining the outcomes of robotic surgery since its recent introduction. As robotic surgery becomes a more common and recognized practice, the costs should ideally eventually decrease with a proportional increase in availability.

Robotic resections seem to be technically feasible and safe, with at least comparable outcomes, as demonstrated by our data and published literature [10]. Wristed instruments, tremor reduction, and high definition 3D views in RA are all of use to surgeons operating in confined spaces, including the pelvis, which can be advantageous when dealing with edematous tissue planes and shortened mesentery in IBD surgery [10]. A robotic platform also offers more precise dissection in embryological planes and preservation of pelvic nerves and helps reduce tissue trauma, leading to reduced complications and quicker recovery as compared to laparoscopy [10].

Multiple trials are looking into the outcomes of robotic surgery worldwide, including ROLARR (UK), REAL (China), ROBOCOSTES (Spain), VANTAGE (Netherlands), and REINFORCE. We aim to continue our data collection prospectively to look at a bigger sample size and as more surgeons are training and performed robotic procedures in IBD as a routine practice.

The limitations of the study include a small sample size owing to the data being from a single center, recent installation of robotic surgery for IBD at our center, and limited surgeons trained in the modality. Learning curve of recently introduced modality has affected factors such as procedure time, conversion rates, procedure costs, and hospital stay. These were also influenced by patient selection based on BMI, surgeon preference, and expertise. The theater costs were done from the available estimate costs from the theater team. For the total costs, the expenditure for each medication consumed or ward-based procedures were not calculated. This is especially relevant in protracted stay, post-operative complications, and readmissions. Only the intraoperative costs for each modality and procedure were available and calculated. We tried to look at the estimated blood loss but could not gather data for all patients due to poor record-keeping. The center does use BMI as a criterion for assessing if it is feasible for a patient to undergo robotic surgery in pre-operative assessment. Patient factors such as race, BMI, and qualitative outcomes were not assessed.

## Conclusions

Robotic surgery showed feasible and comparable prospects in terms of procedure time, conversion rates, hospital stay, complications, and readmission rates in our study. As expected, the average total costs of robotic procedures were significantly higher than laparoscopic procedures at our center. The RA would offer advantages in IBD, which should be studied further in prospective studies, along with long-term qualitative functional outcomes, especially after pelvic surgery, which we aim to look at.
